# Supplementary material for: Prediction of White Matter Hyperintensity in Brain MRI Using Fundus Photographs via Deep Learning
Source: J Clin Med. 2022 Jun 9;11(12):3309. doi: 10.3390/jcm11123309 (PMC9224833; doi:10.3390/jcm11123309)
Supplement: Supplementary file 1 [file jcm-11-03309-s001.zip › jcm-1684779-supplementary.pdf]

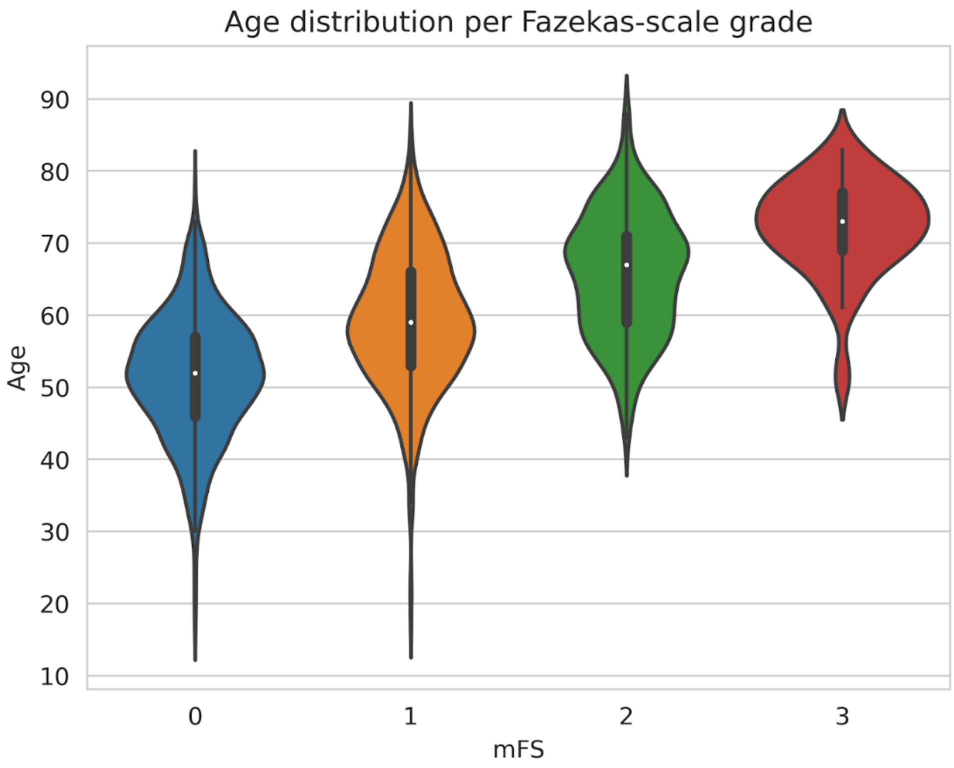

Figure S1. The age distribution of included subjects by modified Fazekas scale grade

Table S1. Assessment of modified Fazekas' score by two stroke neurologists.

	Rater 2				
Rater 1	0	1	2	3	Total
0	894	113	0	0	1007
1	125	524	0	0	649
2	0	4	186	17	207
3	0	0	3	26	29
Total	1019	641	189	43	1892

Table S2. Comparison of Age distribution of each cell in DenseNet-201 confusion matrix

Age (years ± SD)		Predicted class	
		+	-
True Class	+	TP<br>66.3 ± 7.9	FN<br>53.2 ± 9.8
	-	FP<br>59.5 ± 5.4	TN<br>49.2 ± 6.8

SD, standard deviation.

Table S3. Prediction of the presence of WMH (binary classification) using 10-fold cross validation

	DenseNet 201		EfficientNet B7	
	**AUC**	**Accuracy**	**AUC**	**Accuracy**
Fold 0	0.736	0.676	0.712	0.646
Fold 1	0.725	0.678	0.690	0.651
Fold 2	0.750	0.672	0.726	0.653
Fold 3	0.750	0.694	0.718	0.665
Fold 4	0.763	0.685	0.748	0.691
Fold 5	0.734	0.656	0.723	0.661
Fold 6	0.683	0.661	0.692	0.683
Fold 7	0.769	0.708	0.766	0.716
Fold 8	0.687	0.635	0.712	0.665
Fold 9	0.761	0.689	0.756	0.708
Performance average ± SD (95% CI)	0.736±0.030 (0.733-0.739)	0.676±0.021 (0.674-0.678)	0.724±0.026 (0.721-0.727)	0.674±0.024 (0.672-0.676)

AUC, the area under a receiver operating characteristic ; SD, standard deviation.

Table S4. Prediction of the presence of WMH (binary classification) using 5-fold cross validation

	DenseNet 201		EfficientNet B7	
	**AUC**	**Accuracy**	**AUC**	**Accuracy**
Fold 0	0.720	0.658	0.701	0.638
Fold 1	0.746	0.670	0.729	0.659
Fold 2	0.753	0.693	0.734	0.691
Fold 3	0.735	0.687	0.714	0.679
Fold 4	0.715	0.651	0.688	0.646
Performance average ± SD (95% CI)	0.734 ± 0.016 (0.733-0.735)	0.672 ± 0.018 (0.671-0.673)	0.713 ± 0.019 (0.712-0.714)	0.663 ± 0.022 (0.661-0.665)

AUC, the area under a receiver operating characteristic ; SD, standard deviation.

Table S5. Prediction of the Fazekas scale grade (3-class classification) using 10-fold cross validation

	DenseNet 201	EfficientNet B7
	Accuracy	
Fold 0	0.451	0.385
Fold 1	0.440	0.429
Fold 2	0.411	0.389
Fold 3	0.367	0.422
Fold 4	0.451	0.396
Fold 5	0.286	0.352
Fold 6	0.378	0.456
Fold 7	0.467	0.489
Fold 8	0.467	0.333
Fold 9	0.429	0.308
Performance average ± SD (95% CI)	0.414±0.057<br>0.402-0.426	0.396 ± 0.056<br>0.385-0.407

SD, standard deviation.

Table S6. Prediction of the Fazekas scale grade (3-class classification) using 5-fold cross validation

	DenseNet 201	EfficientNet B7
	Accuracy	
Fold 0	0.459	0.414
Fold 1	0.385	0.396
Fold 2	0.354	0.392
Fold 3	0.428	0.367
Fold 4	0.381	0.409
Performance average ± SD	0.401±0.042	0.396±0.018
(95% CI)	0.395 ~0.407	0.393~0.399

SD, standard deviation.
